# An Experimenter's Influence on Motor Enhancements: The Effects of Letter Congruency and Sensory Switch-Costs on Multisensory Integration

**DOI:** 10.3389/fpsyg.2020.588343

**Published:** 2020-12-01

**Authors:** Ayla Barutchu, Charles Spence

**Affiliations:** Department of Experimental Psychology, University of Oxford, Oxford, United Kingdom

**Keywords:** multisensory, auditory, visual, switch cost, letter congruence

## Abstract

Multisensory integration can alter information processing, and previous research has shown that such processes are modulated by sensory switch costs and prior experience (e.g., semantic or letter congruence). Here we report an incidental finding demonstrating, for the first time, the interplay between these processes and experimental factors, specifically the presence (vs. absence) of the experimenter in the testing room. Experiment 1 demonstrates that multisensory motor facilitation in response to audiovisual stimuli (circle and tone with no prior learnt associations) is higher in those trials in which the sensory modality switches than when it repeats. Those participants who completed the study while alone exhibited increased RT variability. Experiment 2 replicated these findings using the letters “b” and “d” presented as unisensory stimuli or congruent and incongruent multisensory stimuli (i.e., grapheme-phoneme pairs). Multisensory enhancements were inflated following a sensory switch; that is, congruent and incongruent multisensory stimuli resulted in significant gains following a sensory switch in the monitored condition. However, when the participants were left alone, multisensory enhancements were only observed for repeating incongruent multisensory stimuli. These incidental findings therefore suggest that the effects of letter congruence and sensory switching on multisensory integration are partly modulated by the presence of an experimenter.

## Introduction

The merging of information from different senses (commonly referred to multisensory integration) can alter, and hence potentially enhance, multisensory information processing. When signals are presented synchronously in different senses, information processing may be facilitated. This includes improvements in response accuracy, learning, memory, and motor performance that have been documented across the lifespan (e.g., Seitz et al., 2006; Shams and Seitz, 2008; Barutchu et al., 2009, 2019a; Flom and Bahrick, 2010; Bremner et al., 2012; Spence, 2013). Many of these multisensory processes also happen to be modulated by attention (e.g., Talsma et al., 2010), prior learnt associations such as in the case of object or letter congruence (e.g., Raij et al., 2000; Molholm et al., 2004; Chen and Spence, 2010; Cox and Hong, 2015), and other factors, such as attention switching between the senses (e.g., Otto and Mamassian, 2012). However, to date, we are aware of no study that has specifically investigated how attention, sensory switching, and learnt associations interact with one another to influence multisensory processes.

Various multisensory processes and related neural mechanisms are modulated by attention (e.g., Alsius et al., 2005; Fairhall and Macaluso, 2009; Talsma et al., 2010; Botta et al., 2011; Talsma, 2015; Barutchu et al., 2019b; see Spence and Soto-Faraco, 2020, for a review). The research shows that multisensory stimuli are more likely to be integrated if they happen to be presented from an attended (as compared to a relatively less attended) location, or if the stimuli are themselves salient enough to capture a participant's attention in a bottom-up manner (Talsma et al., 2010). Several studies also suggest that if attention happens to be divided across tasks, then multisensory illusions, such as the McGurk effect, may be significantly weakened (Alsius et al., 2005). Another factor that may also influence attention and, in turn, multisensory processing that has not been investigated previously (in the latter context) is the presence of an experimenter in the testing room. Indeed, research conducted over the last decade has shown that various social influences, including joint attention, can alter both visual and audiovisual task performance, even under those conditions where the “co-actor” happens not to be visible to the participant (e.g., Atmaca et al., 2011; Dittrich et al., 2017; Gregory and Jackson, 2017; Wahn et al., 2017; Hobeika et al., 2020). For example, Wahn et al. (2017) showed spatial localization of sensory stimuli was significantly slower with spatially incongruent audiovisual presentations when the participant was alone then when two participants performed the same task simultaneously in close proximity. An experimenter, unlike a co-participant in a joint attention study, is in a position of authority and responsibility, while at the same time being less involved in the task at hand on a moment-by-moment basis. Thus, the presence of an experimenter could also alter conformity and obedience to task instructions as well as levels of motivation (e.g., Sherif, 1935; Asch, 1956; Milgram, 1965). This, in turn, might be expected to lead to an up-regulation of vigilance, attention and, in turn, motivation, during experimental testing. Indeed, under certain experimental conditions, the presence of an experimenter has previously been shown to improve the accuracy of signal detection in adults (Putz, 1975), and performace on attention taks in hyperactive boys (Gomez and Sanson, 1994). At the same time, however, the presence of an experimenter in the testing room may also be expected to draw the participant's attention away from the target stimuli, and hence hinder, and thus possibly alter, task performance as a result (Risko and Kingstone, 2011; Belletier et al., 2015; Belletier and Camos, 2018).

Multisensory processes are also influenced by attentional switches between the sensory modalities—historically also commonly referred to as the “Modality Shift Effect” (MSE) (e.g., Sutton et al., 1961; Cohen and Rist, 1992). To be consistent with the broader literature, we define “switching” as a change in stimulus type across consecutive trials; Audiovisual stimuli are defined as a different type of stimulus despite the fact that they share an overlapping component with each of the unisensory auditory and visual stimuli. Switching between different unisensory systems results in slower RTs (following the switch), and that, in some clinical populations, sensory attention networks are less flexible, and hence prone to exhibiting larger modality switch costs than in the general population (e.g., Sutton et al., 1961; Ferstl et al., 1994; Hanewinkel and Ferstl, 1996; Spence et al., 2001; Turatto et al., 2002; Lukas et al., 2010; Harrar et al., 2014; Innes and Otto, 2019; Liu and Otto, 2020; Shaw et al., 2020). It is generally assumed that switch costs, both within and across the senses, typically observed in cueing and task switching paradigms, reflect shifts in attention across or within the senses and that such shifts in attention can degrade/slow information processing (e.g., Lukas et al., 2010; Longman et al., 2014; Lin and Carlile, 2015; Swainson et al., 2017).

In some influential studies, it has been proposed that sensory switching, together with race-models and changes in RT variability under multisensory conditions may explain multisensory motor speed enhancements (Otto and Mamassian, 2012; Otto et al., 2013). The latter researchers used the classic simple audiovisual detection task with random presentations of auditory, visual, and audiovisual stimuli in order to show that switch costs are higher when switching between different unisensory modalities, thus inflating multisensory RT gains. They proposed that once switch costs are accounted for, multisensory enhancements can be explained by “statistical facilitation” in line with traditional race models (see also the introduction to Experiment 1 below). If a motor response is always initiated by the faster of two signals then, naturally, reaction times (RTs) to multisensory signals will be faster than to unisensory signals. Alternatively, however, according to “co-activation” models, at some point during information processing, neural network signals from different sensory systems “pool” to reach a response initiation criterion faster, thus resulting in faster RTs (Miller, 1982, 1986, 1991). Indeed, studies have consistently demonstrated that multisensory enhancements may be too large to be explained merely by “statistical facilitation” in adults using Miller's test of inequality. The latter is calculated by adding the probabilities along the distribution of the unisensory RTs and showing that the summed probabilities of the unisensory signals cannot predict the fastest responses of the multisensory RT distribution. However, in the classic detection paradigm with random stimulus presentation, the serial order of auditory and visual signals influence each other; that is, RTs for auditory and visual signals are slower following a sensory switch, when compared to repeat conditions. As yet, however, it is unknown how unisensory and multisensory switch costs are influenced by letter congruence and other experimental factors, such as, for instance, the presence of an experimenter in the testing room. Both the novelty of incongruent letters, and the presence of an experimenter, are likely to modulate attention, and sensory switching is associated with shifts in attention. Therefore, it is likely that sensory switch costs will be modulated by letter congruence as well as by the presence of the experimenter.

The two experiments reported here are the first to investigate the influence of prior learnt relations, and sensory switching, on multisensory processing. The first experiment investigated RT enhancements in a simple speeded detection paradigm using signals without any prior specific learnt associations. In particular, a red circle was coupled with a pure tone, and auditory, visual, and audiovisual stimuli were presented in a random sequence. Gain measures for repeat and stimulus switch conditions were compared, with the hypothesis being that multisensory enhancements would be significantly greater for switch than for repeat conditions. These processes were modulated by the presence of the experimenter in the testing room. We then ran a second study (Experiment 2) using a simple detection task with random unisensory and multisensory presentations of the letters “b” and “d” of the English alphabet (i.e., the associated graphemes and phonemes) in order to investigate whether we could replicate these unexpected findings with prior learnt relations. Specifically, the participants in our second study had to respond to all letters (i.e., including multisensory stimuli, regardless of whether or not they were congruent). If anything, the presence of the experimenter is likely to increase participants' vigilance, motivation and attention levels. Therefore, for both experiments, larger multisensory enhancements were predicted when the participants were monitored than when they were left alone by themselves in the testing room.

## Experiment 1

The original aim of the first experiment had been to investigate unisensory and multisensory switching using novel associations in the typical simple multisensory detection paradigm with random presentations of auditory, visual, and audiovisual stimuli. Initially, the two experiments reported here were designed to replicate prior findings by Otto and Mamassian, and the experiment was conducted with the participants left alone in a quiet isolated room (e.g., Otto and Mamassian, 2012). However, unusually high RT variability was noted in this first attempt compared to the previous studies by Barutchu et al. in which the experimenter was seated in the room with the participant, the most obvious explanation being the absence of the experimenter from the testing room (e.g., Barutchu et al., 2009, 2018). Within the multisensory literature, in studies that test children and clinical patients, for reasons of practicality, participants are often closely monitored with the experimenter or caregiver typically seated in the room (e.g., Barutchu et al., 2009, 2018; Bremner et al., 2012). However, in adult studies, participants are typically left alone during the testing phase of any experiment. A separate body of research has demonstrated that multisensory processes can also be influenced by the presence of another person jointly performing the task (Wahn et al., 2017; Hobeika et al., 2020).

To the best of our knowledge, no one has yet explicitly investigated the effects of the presence of an experimenter on multisensory information processing. Therefore, we re-ran the studies replicating prior experimental conditions, but with the experimenter now seated in the room, out of sight of the participant, with the idea being subtly to encourage the participants to maintain their vigilance and attention during the task (e.g., Barutchu et al., 2009, 2018). Understanding the influence of the experimenter is undoubtedly an important contribution to the literature as multisensory processes in everyday life typically occur in social situations, whereas the experimental participant is often isolated from any social interaction (Soto-Faraco et al., 2019). Here, we report two studies with the participants either left alone or else monitored. We predicted slower RTs following a sensory switch, and less RT enhancement for repeat than for switch stimuli, and that these processes will be affected by the presence of the experimenter.

## Materials and Methods

### Participants

Both multisensory effects in simple speeded detection paradigms and switch costs are generally associated with large effect sizes (e.g., Barutchu et al., 2009, 2018). However, here we took the more conservative approach and assumed moderate effect sizes. Using G-power, for a within-participant design with nine repeated measures, for a moderate effect size = 0.4, power = 0.8, and set at alpha = 0.05, the recommended sample size is 7 (Faul et al., 2007). Nevertheless, we recruited 10–15 participants per experimental group in order to improve the reliability of the samples (Cumming, 2013). Initially, 15 healthy young adults aged between 20 and 31 years (*M* age = 24 years, 9 males, 6 females) were recruited in the “alone” condition. Following our failure to replicate past studies (e.g., Barutchu et al., 2009, 2018), we repeated the study under “monitored” conditions. To the best of our knowledge, the comparison between alone and monitored conditions is the first of its kind, meaning that there is no prior estimate of effect size. Thus, we adopted the alternative systematic approach of sampling and re-analyzing every 4 cases (note that, in general, motor multisensory enhancements are very reliable and observable at an individual level). Indeed, under “monitored” conditions, we were able to replicate previous studies with fewer than 8 participants; however, we failed to see significant differences between the alone and monitored groups. Therefore, we recruited an additional 4 cases, which still yielded a very small effect size of <0.1 for some measures in the alone and monitored group comparisons (i.e., RTs, note that over 400 participants would be required to show a significant difference for an effect size of 0.1, which is obviously well outside the scope of such a psychophysical study). Thus, in total, we recruited 12 health young adults between 21 and 32 years of age (*M* = 25 years, 3 males, 9 females), who were allocated to the “monitored” condition. Note also that a group size of 12 is wholly comparable with other multisensory psychophysical studies we were aiming to replicate that reported participant numbers as low as 10 (e.g., Otto et al., 2013). None of the participants reported any prior history of neurological of psychiatric conditions. The participants were paid £10 for taking part in a study that took ~1 h to complete.

All of the participants provided informed consent prior to taking part in the study, and all procedures were ethically approved and strictly adhered to the guidelines of the Inter Divisional Medical Sciences Research Ethics Committee, University of Oxford.

### Stimuli

The participants were presented with auditory (AT), visual (VT), and audiovisual target stimuli (AVT). The visual stimulus was a red circle (2.1 cm radius) presented at the center of a 17-inch monitor. The auditory stimulus was a 500 Hz pure tone (5 ms rise and fall time) presented from two loudspeakers, one positioned on either side of the monitor. The auditory stimulus was presented with equal intensity to both ears, measured at 75 dB at the participant's ear (note that this set-up led to the sound appearing to come from the center of the screen; i.e., from the same apparent location as the visual target). For audiovisual stimuli, the auditory and visual stimuli were presented simultaneously. An oscilloscope was used to confirm the synchronization of the auditory and visual signals (a jitter of <1 ms was detected). The stimuli were all presented for 100 ms. There were 6 blocks of 180 stimuli (~130 presentation per stimulus type and switch condition). The interstimulus interval (ISI) between successive stimuli varied randomly between 1,250 and 2,250 ms. The duration of each block of trials was ~5 min.

### Procedure

The participants were seated in a quiet dimly-illuminated room and positioned centrally at a distance of ~75 cm from the computer monitor (exactly the same room and lighting conditions were used for both the alone and monitored condition). The same testing environment was used for the alone and monitored condition. In the alone condition, the experimenter only remained in the room for the practice trials (a maximum of 20 trials in order to ensure that the participant understood the task instructions), and returned once all of the blocks of stimuli had been presented. This meant that the participants were isolated for a total of ~40 min. In the monitored condition, the experimenter remained in the testing room at all times and was seated ~1 meter and ~110 degrees from fixation (i.e., to the side and out of the participant's line of peripheral sight, but in a position able to monitor their direction of fixation and movements). The experimenter monitored the participant throughout the task to ensure that they maintained fixation on the screen. The participants were instructed to respond to all of the target stimuli (i.e., AT, VT, and AVT stimuli) using a response pad with a finger of the right hand. Initially, the participants were presented with a fixation cross (size = 0.5 cm) from the center of the screen for 500 ms followed by a random sequence of equiprobable AT, VT, and AVT stimuli, thus, the switch between the AT, VT, and AVT stimuli was also random. There were no significant differences between the numbers of switch trials for each stimulus type. Before the initiation of the first block of trials, all of the participants were encouraged to take a break of at least 1–5 min between each block of trials. In the monitored condition, the experimenter asked the participants how they were doing and told them that they could initiate the next block of trials whenever they were ready. In both the alone and the monitored condition, the participants determined the duration of the breaks and self-initiated the blocks when ready. The total duration of the experiment, including breaks, was ~40–45 min for both the monitored and the alone experimental conditions.

### Design and Data Processing

Both of the experiments reported here used a simple detection paradigm, whereby the participants had to respond to all of the target stimuli as rapidly and accurately as possible. Therefore, an error is defined as a failure to respond to a stimulus (i.e., all errors are omissions).

A 2(testing condition: alone and monitored) x 3(stimulus type: AT, VT, and ATVT) × 3(switch type: preAT, preVT, and preAVT) mixed design was used. The between-group measure had 2 levels: the participants were either “monitored” by the experimenter or else “alone.” The two repeated measures had 3 levels each: stimulus type (AT, VT, and AVT) and switch type (preAT, preVT, and preAVT). The term “pre” refers to the previous stimulus in the sequence of trials, which is indicative of the switch type (e.g., an AT with a preAT is a repeat condition, while an AT with a preVT is a switch condition whereby AT stimulus was preceded by a VT stimulus).

For all of the studies reported here, for each individual, only motor responses >100 ms and <3 SD below the means were accepted as correct motor responses (RTs) and included in the analyses reported below. Less than 2% of RTs were rejected based on these exclusion criteria. For each experimental (alone and monitored) and switch (repeat and switch) condition, moving averages were calculated by averaging the RTs across 10 consecutive trials moving in steps of one. In addition, for each participant, the individual RT coefficient of variation was calculated by dividing the standard deviation (*SD*) of RTs by the mean (μ) RT (*Cv* = *SD*/μ). Differences between the stimulus type, switch conditions, and experimental conditions for percentage error rates, RTs, and the coefficient of variation (*Cv*) were assessed using a series of 2(testing condition: monitored and alone) x 3(stimulus type: AT, VT, and AVT) x 3(switch type: preAT, preVT, and preAVT) mixed Analysis of Variance (ANOVA).

Multisensory enhancement or “gain” measures were calculated by subtracting the RT for the multisensory stimulus from the faster of the two mean unisensory RTs. Therefore, a positive gain value represents faster RTs for multisensory stimuli, whereas a negative value indicates a cost associated with responding to multisensory (as compared to unisensory) stimuli. Multisensory gain measures were analyzed using a 2(switch condition: repeat and switch) x 2(testing condition: monitored and alone) mixed ANOVA. In addition, for each stimulus and switch condition, cumulative density functions (CDFs) and Miller's test of the race-model inequality were also calculated at an individual level (see Miller, 1982, for details). Violations of the race-model were assessed using a series of 2(AVT vs. AT+VT CDF) × 10 (probabilities) ANOVAs. Since violations of race models only concern the fastest RTs, planned contrasts were applied to the faster end of the CDF distribution (i.e., for probabilities of 0.55 and below) to assess whether the AVT CDF was significantly faster than the AT+VT CDF (whereby the probabilities of the RTs along the CDF are added, see Miller, 1982).

Switch costs were also calculated by subtracting the switch conditions from the repeat conditions with negative values reflecting a cost in RT following a switch (i.e., slower RTs), while a positive value represents a gain in RT following a switch (i.e., faster RTs). A 6(switch conditions: AT-preVT, AT-preAVT, VT-preAT, VT- preAVT, AVT-preAT, and AVT-preVT) × 2(testing condition: alone and monitored) mixed ANOVA was used to assess switch costs across the experimental conditions.

All significant interaction effects were followed-up with simple effects analyses, and Bonferroni adjustments were applied to multiple *post-hoc* comparisons, where appropriate.

## Results

As expected, the overall percentage of errors on the simple detection task was low, averaging below 10% mean errors for all stimulus conditions, and significantly lower when the experimenter was present in the room (*M* = 2.74%, *SE* = 0.74) than when the participants were left alone (*M* = 5.25%, *SE* = 0.47) (see Appendix A for a detailed outline of the accuracy analysis).

### Reaction Times

**Figure 1:**
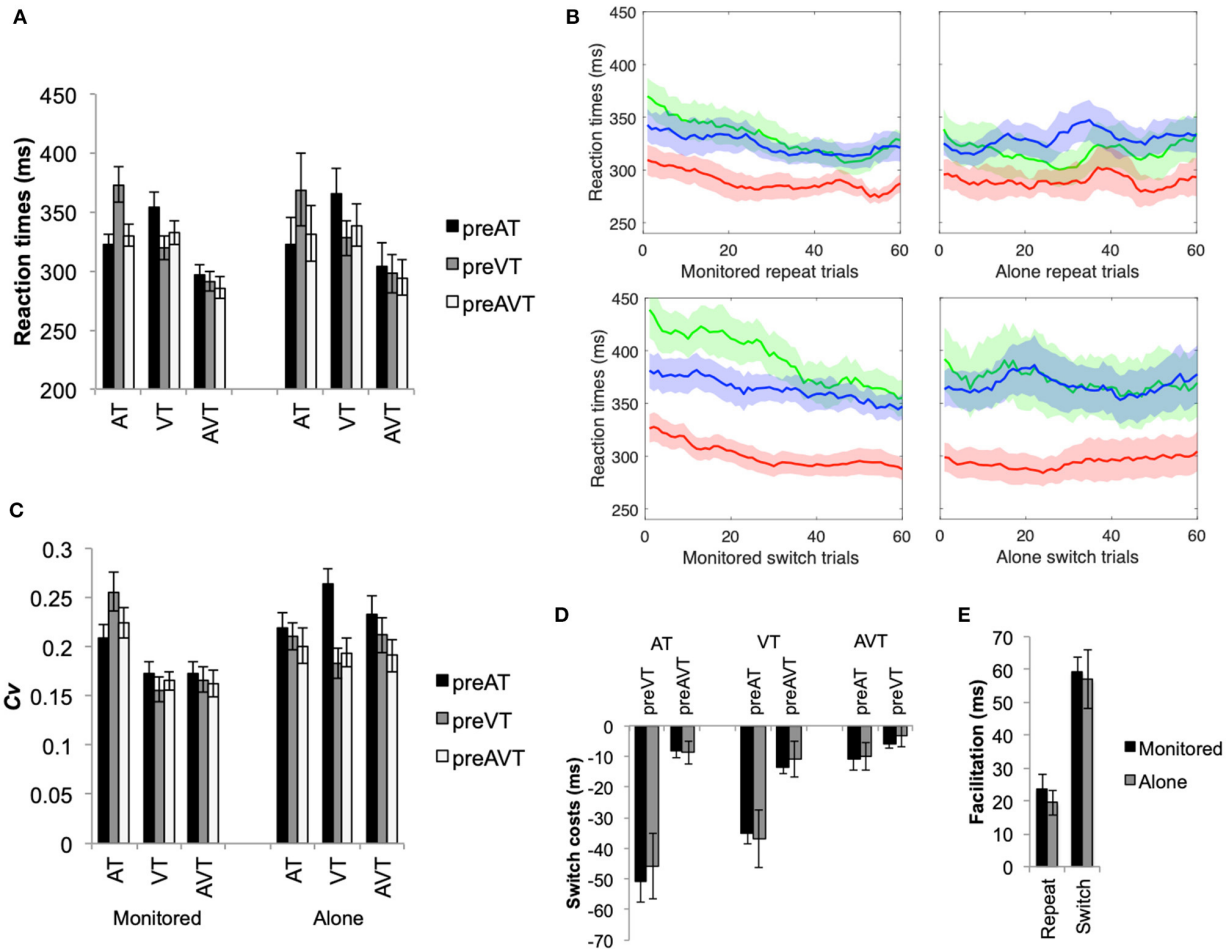
**(A)** Mean RT (+SEM) for the auditory (AT), visual (VT), and audiovisual (AVT) stimuli, switch (pre-AT, pre-VT, and pre-AVT) and experimental (monitored and alone) conditions. **(B)** Moving averages in steps of 1 averaged over 10 RTs for the first 60 trials for repeat and switch conditions and the monitored and alone conditions (green = AT, blue = VT, and red = AVT). Note that moving averages and SEMs were calculated for the first 60 trials only, as errors of omission were as high as 18% for some participants in the alone condition (i.e., there are not enough RTs from some participants). **(C)** Coefficient of Variation (+SEM), and **(D)** switch costs (+SEM) for auditory visual and audiovisual stimuli and switch conditions (i.e., switch cost = repeat—switch condition). **(E)** Mean multisensory gain in ms (+SEM) for the switch and the alone and monitored experimental conditions. Note that the switch gain represents the difference between the faster of the unisensory switch conditions and the faster of the multisensory switch conditions.

As predicted, multisensory RT facilitation was observed; mean RTs (see Figure 1A) were significantly faster for audiovisual stimuli than for the unisensory stimuli, *F*_(2, 100)_ = 45.80, *p* < 0.001, η^2^ =0.65 (*p* < 0.001 for both follow-up main effects analyses comparing AVT with AT and VT). The main effect of switching, *F*_(2, 100)_ = 12.86, *p* < 0.001, η^2^ = 0.34, and the interaction between stimulus type and switching, *F*_(4, 100)_ = 40.15, *p* < 0.001, η^2^ = 0.62, were also significant. The main effect for group (i.e., monitored vs. alone) was, however, not significant, *F*_(1, 24)_ = 0.05, *p* = 0.82, η^2^ = 0.002. Follow-up simple effects analyses revealed significantly slower RTs when switching between unisensory stimuli (*p* < 0.002) as compared to the repeat conditions (see Figure 1A). By contrast, RTs for the multisensory stimuli were not affected by switching from VT stimuli (*p* = 0.40), with only the small increase in RT when switching from an AT stimulus reaching significance (*p* = 0.009). Furthermore, when switching from AVT, the RTs for AT and VT did not differ significantly (*p* > 0.9). There were no other significant main and interaction effects in the mean RT data (*p* > 0.7 for all).

The moving averages with SEMs (see Figure 1B) were also calculated as a descriptive to visually observe the trend of RTs over the course of the experiment. As can be observed, RTs for the multisensory stimuli are consistently faster than the unisensory stimuli throughout the entire duration of the task for both the monitored and the alone conditions. Additionally, RTs tended to speed-up during the course of the study, particularly in the monitored condition. Interestingly, RT variability across individuals (i.e., shaded areas in Figure 1B depicting SEMs) was higher and more likely to overlap across the unisensory (AT and VT) and the multisensory (AVT) conditions when the participants were left alone than when the experimenter was present in the testing room. Indeed, the variability of RTs across individuals was significantly higher in the alone than in the monitored conditions (see Appendix C for additional analyses).

The variability of individual participants' RTs was also assessed (see Figure 1C). An analysis of the Coefficient of Variation (*Cv*) of RTs revealed that overall variability was significantly higher for auditory stimuli than for visual or audiovisual stimuli, *F*_(2, 100)_ = 36.00, *p* < 0.001, η^2^ = 0.59. The stimulus type by switch condition interaction was also significant, *F*_(4, 100)_ = 12.46, *p* < 0.001, η^2^ = 0.33. In particular, switching between unisensory auditory and visual stimuli significantly increased RT variability for unisensory stimuli with the largest observed increase in RT variability being documented for AT stimuli when switching from VT stimuli (*p* < 0.01 for all *post-hoc* pairwise simple effects comparisons). Switching from an audiovisual stimulus to an unisensory stimulus also led to a significant increase in RT variability. By contrast, RT variability for the AVT stimulus was not significantly affected by the various switch conditions (*p* > 0.1 for all). There were no other significant main and interaction effects for C*v* (*p* > 0.1 for all).

### Switch Costs

This simple detection task required participants to respond to all target stimuli using the same button press, therefore only the stimulus modality changed across consecutive trials. Switch costs were calculated by subtracting the switch conditions from the repeat conditions (i.e., switch costs = repeat RT—switch RT, see Methods section for details). Nevertheless, switch costs increased significantly when switching between unisensory stimuli as compared to when switching between unisensory and multisensory stimuli (see Figure 1D), *F*_(1, 25)_ = 24.74, *p* < 0.001, η^2^ = 0.50. Follow-up main effects analyses revealed that unisensory switch costs were significantly larger than multisensory switch costs (*p* < 0.003 for all comparisons), with the highest (significant) switch cost being documented for AT stimuli when switching from a VT stimulus (*p* < 0.001). Switch costs for the multisensory stimuli did not differ significantly when switching between unisensory and multisensory stimuli (*p* > 0.4 for all). There were no other significant main and interaction effects for switch-costs (*p* > 0.7 for all).

### Multisensory Enhancements

**Figure 2:**
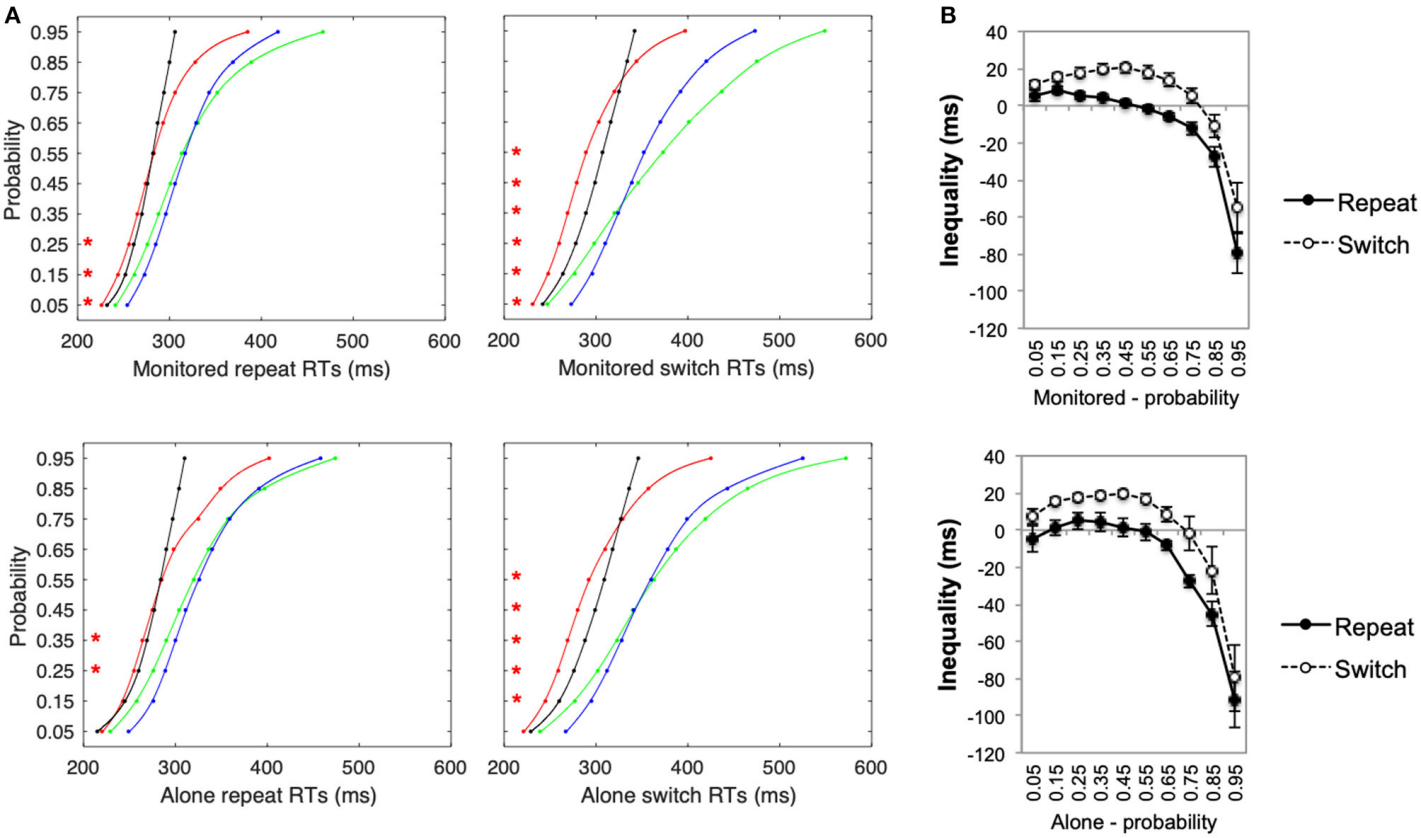
Experiment 1 **(A)** Cumulative probability functions (CPFs) for auditory (green), visual (blue), multisensory stimuli (red), and for the summed probability of the unisensory stimuli (AT+VT CDF—i.e., test of inequality, black) for repeat and switch trials under alone and monitored experimental conditions. **(B)** Inequality calculated as the difference between the AVT CDF (red line) and the AT+VT CDFs (black line) for repeat and switch trials under alone and monitored experimental conditions. Red *p < 0.05.

Multisensory gains in RTs (see Figure 1E, whereby gain = faster of unisensory—multisensory condition, thus positive values = faster RTs for multisensory stimuli) were significantly lower for repeat than for switch trials, *F*_(1, 25)_ = 43.85, *p* < 0.001, η^2^ = 0.64. Switching between sensory target stimuli slowed participants' responses and increased the variability of their RTs for unisensory, but not for multisensory, stimuli thus amplifying the observed multisensory facilitation in the switch conditions. The difference in multisensory RT gains (see Figure 1E) between the alone and the monitored condition was not significant, *F*_(1, 25)_ = 0.03, *p* = 0.86, η^2^ = 0.001. Furthermore, as switch costs have a significantly greater effect on unisensory than multisensory stimuli, more violations of the race-model of inequality were observed for switch than for repeat conditions (see Figure 2). Nevertheless, in the monitored condition, significant race-model violations were still observed in the fastest RTs for repeat conditions; a 2(AVT vs. AT+VT CDF) ×10(probabilities) ANOVA revealed a significant interaction, *F*_(9, 99)_ = 41.64, *p* < 0.001, η^2^ = 0.79. Follow-up planned comparisons revealed significant violations for the monitored repeat stimuli 0.05 to 0.15 probability (*p* < 0.03 for all planned *post-hoc* contrasts). For the monitored switch conditions, significant violations ranged from a probability of 0.05 to 0.55, *F*_(9, 99)_ = 35.39, *p* < 0.001, η^2^ = 0.76 (*p* < 0.006 for all planned *post-hoc* contrasts). In the alone repeat conditions, there were no significant violations of the race model for the fastest RTs, *F*_(9, 126)_ = 20.75, *p* < 0.001, η^2^ = 0.59; race model violations were observed only for the 0.35 and 0.45 probabilities (*p* < 0.03 for both). For the alone switch conditions, significant race-model violations ranged from 0.15 to 0.55 probabilities, *F*_(9, 126)_ = 23.70, *p* < 0.001, η^2^ = 0.63 (*p* < 0.002 for all planned *post-hoc* contrasts). Although measured comparisons between the alone and monitored condition do not differ significantly, Experiment 1 nevertheless reveals that the presence of the experimenter in the testing room influences race-violations. Thus, inconsistencies across multisensory studies could potentially be explained by the presence of an experimenter in the testing room.

## Discussion

In Experiment 1, a simple speeded detection paradigm was used to demonstrate that RTs are significantly faster for multisensory than for unisensory stimuli under both repeat and switch conditions. This multisensory advantage could not be explained simply as a result of increases in an individual's RT variability for multisensory stimuli. The RT variability for multisensory stimuli was not significantly affected by stimulus switching, unlike the unisensory stimuli, which not only slowed down but showed an increase in RT variability (i.e., *Cv*) with switching. The magnitude of the multisensory gain was thus amplified on switch, but not on repeat, trials. Nevertheless, Miller's test of inequality was violated under both repeat and switch conditions in the monitored condition. However, violations were not observed for the fastest RTs when the participants were alone in the testing room. This suggests that multisensory enhancement effects are partly dependent on the experimental testing conditions (specifically, the presence vs. absence of an experimenter in the testing room).

Multisensory enhancements were observed for both repeat and switch conditions throughout the entire sequence of trials. As expected, switching between unisensory stimuli slowed RTs significantly (e.g., Cohen and Rist, 1992; Turatto et al., 2002; Otto and Mamassian, 2012, 2016), and increased their variability. RTs to multisensory stimuli, on the other hand, were not affected by sensory switching. When a multisensory stimulus (AVT—i.e., tone with red circle in this case) was preceded by a unisensory auditory (AT—tone) or visual (VT—red circle) stimulus, there was always one component of the target that repeated, in turn, potentially eliminating switch costs. Since switch costs are only amplified for unisensory stimuli, this increases the observed overall multisensory gain for the switch as compared to the repeat conditions. Nevertheless, in Experiment 1, we still observed significant multisensory enhancements for both repeat and switch conditions under the monitored conditions.

Given the unexpected nature of the experimenter effect reported in Experiment 1, we aimed in Experiment 2 to replicate this finding and show that the presence of the experimenter (in the room) can indeed modulate multisensory processes using stimuli with well-learnt associations, i.e., letters instead (i.e., the graphemes and phonemes for the letters “b” and “d”).

## Experiment 2

Experiment 2 aimed to replicate and further investigate the effects of sensory switching of learnt stimuli on multisensory facilitation. Prior learning and letter congruence have been shown to exert an influence over various multisensory processes (e.g., Raij et al., 2000; Molholm et al., 2004; Sinnett et al., 2008; Delogu et al., 2009; Chen and Spence, 2013, 2017, 2018; Downing et al., 2014). Multisensory associations can also be learnt faster (e.g., when a coincidental novel sound, coupled with a visual stimulus, are both defined as targets via feedback) (e.g., Fifer et al., 2013); and both well-learnt semantically congruent associations and unfamiliar pairings can lead to multisensory enhancement (e.g., Miller, 1982; Giard and Peronnet, 1999; Molholm et al., 2004). However, expectations and context can alter multisensory integration (e.g., Sinnett et al., 2008; Gau and Noppeney, 2016; Shepherdson and Miller, 2016; Barutchu et al., 2018). For example, Barutchu et al. (2018) have demonstrated significant multisensory enhancements to incongruent multisensory stimuli (i.e., comparable multisensory RT facilitations for audiovisual presentations of both congruent “chirping birds” and incongruent “barking birds,” and vice versa) when the learnt associations are not relevant to the task at hand. However, it is unknown how such learnt associations interact with sensory switching, and whether they are influenced by the presence of an experimenter. Given that all three factors can influence attention, it is hypothesized that they are likely to interact with each other to influence multisensory integration. Therefore, in Experiment 2, the letters “b” and “d” were used because of their phonetic and graphemic similarity. The incongruence of the letters was task-irrelevant, in that the participants were asked to make the same simple motor response to all stimuli (i.e., unisensory and multisensory stimuli no matter whether or not they were congruent). Given that in the second experiment the congruence of the stimuli was not relevant to the task at hand, it was hypothesized that multisensory enhancements and switch costs would be similar for congruent and incongruent multisensory stimuli (Barutchu et al., 2018). Multisensory enhancements were also expected to be higher when the experimenter was seated in the room with the participant.

## Materials and Methods

### Participants

Seventeen participants volunteered for the alone condition, one of whom was excluded due to high error rates (over 2.5 SD above the mean) on the detection task, leaving 16 participants in the final analyses reported below (age range = 19–31 years, *M* age = 25 years 5 months, 11 males and 5 females). In addition, 12 healthy young adults volunteered for the monitored condition. One participant was excluded due to high error rates (over 2.5 SD above the mean) on the detection task (age range = between 21 and 31 years, *M* age = 25 years 6 months, 4 males and 7 females). All of the participants either spoke English as a first language or else had started learning English during their early childhood. Participants reported no prior history of neurological of psychiatric conditions. They were paid £10 per hour of their participation.

### Stimuli and Procedure

The stimuli for Experiment 2 included auditory and visual presentations of the lowercase letters “b” and “d” (in bold Arial font 72) and their respective phonemes (i.e., /b/ and /d/ enunciated by a mature female). Black letters on a white background were presented in the center of the screen for 200 ms. Letters were presented as auditory (AT), visual (VT), audiovisual congruent (AVT-c), and audiovisual incongruent stimuli (AVT-ic). The participants had to respond by pressing a response button to all stimuli as rapidly and accurately as possible.

The AT, VT, AVT-c, and AVT-ic stimuli were presented randomly with equal probability in blocks of 240 stimuli. Each block lasted for ~7 min. In the alone condition, the participants were presented with 8 blocks of stimuli. In the monitored condition, the participants were presented with 6 blocks of stimuli. The additional blocks in the alone condition did not affect the mean RTs or the outcomes of the study. Nevertheless, for both experimental conditions, only the first 6 blocks were included in the final analyses reported below for consistency.

### Data Analyses

Experiment 2 also used a simple detection paradigm, whereby participants had to respond to all stimuli as fast and accurately as possible (thus, all errors are errors of omission).

A 2(testing condition: alone and monitored) × 4(stimulus type: AT, VT, AVT-c and AVT-ic) × 4(switch type: preAT, preVT, preAVT-c, and preAVT-ic) mixed design was used. In line with Experiment 1, the between-group measure had 2 levels: the participants were either “monitored” by the experimenter or “alone.” The two repeated measures each had 4 levels: stimulus type (AT, VT, AVT-c, and AVT-ic) and switch type (preAT, preVT, preAVT-c, and preAVT-ic).

Percentage error rates, RTs, and *Cv* measures were analyzed using a series of 2(experimental conditions: alone and monitored) × 4(stimuli type: AT, VT, and AVT-c, and AVT-ic) × 4(switch condition: preAT, preVT, preAVT-c, and preAVT-ic) mixed ANOVAs. Switch conditions were analyzed using a 2(experimental conditions: alone and monitored) × 12(switch type: see **Figure 4B** for a list of conditions) mixed ANOVA. Multisensory enhancements were assessed using a 2(experimental conditions: alone and monitored) × 2(switch: repeat and switch) × 2(congruency: congruent and incongruent) mixed ANOVA.

Miller's test of inequality for experimental (alone and monitored), congruency (congruent and incongruent), and switch (repeat and switch) conditions was statistically assessed using a series of separate 2(stimuli: AVT CDF and AT+VT CDF) × 10(probabilities) repeated measured ANOVAs followed by planned contrasts for the faster RTs with probabilities ≤0.55. All other stimulus parameters, experimental procedures, and all analysis procedures were the same as in Experiment 1.

## Results

Preliminary analyses revealed that compared to the repeating stimuli, switching between stimuli within the same modality (i.e., switching between unisensory graphemes or phonemes) did not affect either response accuracy or RTs. Therefore, unisensory responses for “b” and “d” were collapsed and only the four different stimulus types (i.e., AT, VT, AVT-c, and AVT-ic) were analyzed further.

As expected, accuracy on the simple detection task was high (statistical analyses of the accuracy data are presented in Appendix B).

### Reaction Times

**Figure 3:**
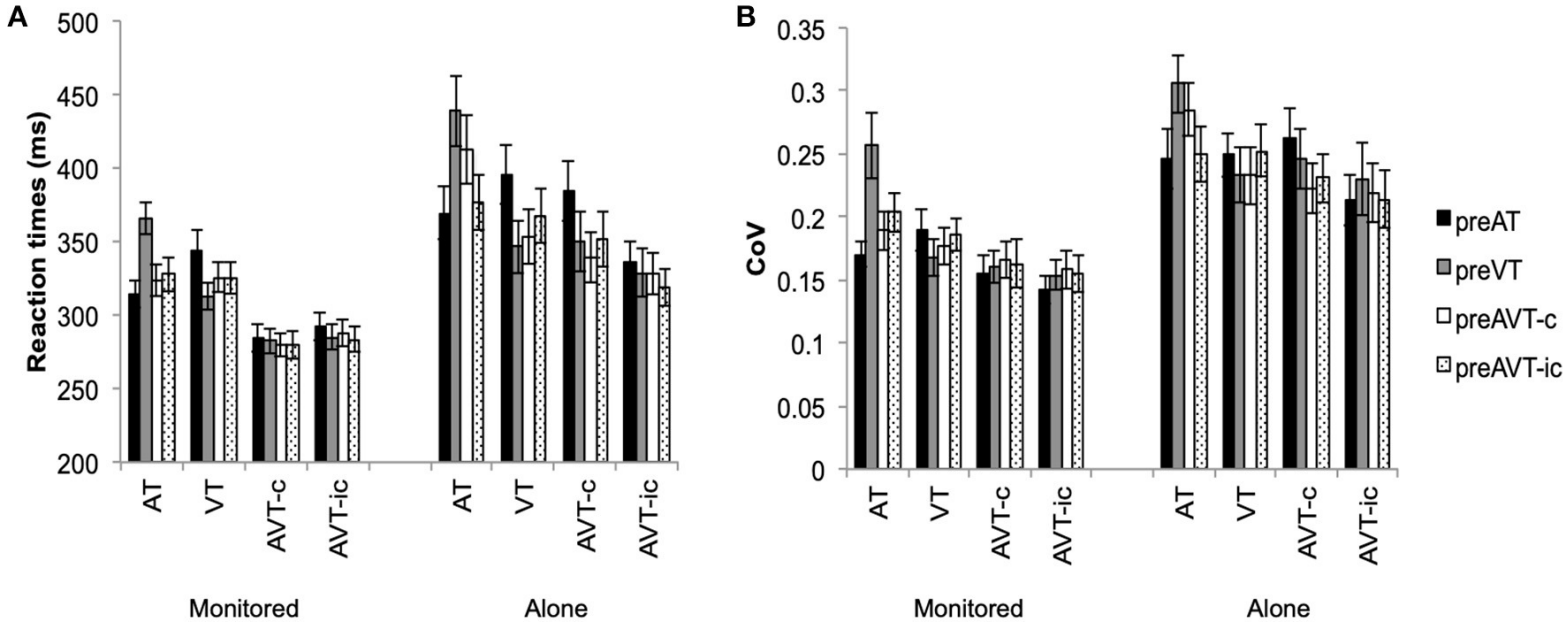
Experiment 2 **(A)** Mean RTs (+SEM) and **(B)** mean Coefficient of Variation (+SEM) for auditory (AT), visual (VT), audiovisual congruent (AVT-c), and audiovisual incongruent (AVT-ic) stimuli and switch conditions.

**Figure 4:**
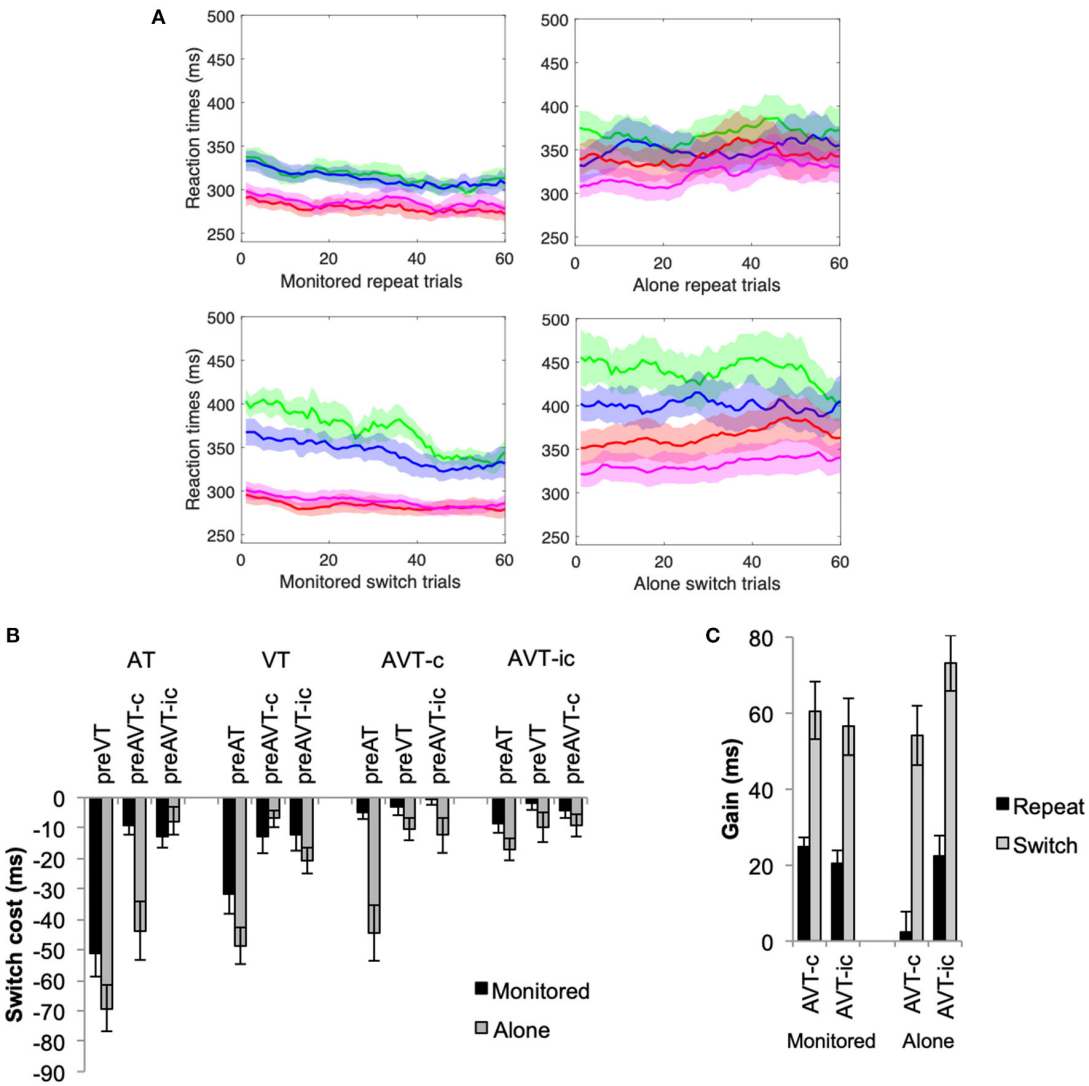
Experiment 2 **(A)** Moving averages of RTs (1 step of 10 trials) for the repeat and switch conditions for the AT (green), VT (blue), AVT-c (red), and AVT-ic (magenta) stimuli. **(B)** Mean switch costs = repeat—switch condition (+SEM), and **(C)** mean MS gain (+SEM) for stimuli, switch, and monitoring conditions.

Consistent with the results of Experiment 1, motor enhancements were observed for both repeat and switch trials (see Figures 3, 4). Mean RTs were significantly faster for the multisensory AVT-c and AVT-ic stimuli than for the unisensory AT and VT stimuli, *F*_(3, 225)_ = 57.14, *p* < 0.001, η^2^ = 0.70. The main effect for switch was also significant whereby switching from the unisensory AT and VT stimuli resulted in slower RTs, *F*_(3, 225)_ = 7.49, *p* < 0.001, η^2^ = 0.23. Mean RTs were slower in the alone than in the monitored condition, *F*_(1, 25)_ = 6.08, *p* = 0.02, η^2^ = 0.20. The two-way interactions between stimulus type and experimental condition, *F*_(3, 225)_ = 6.67, *p* < 0.001, η^2^ = 0.21, stimulus type, and switch condition, *F*_(9, 225)_ = 33.66, *p* < 0.001, η^2^ =0.57, and the three-way interaction between stimulus type, switch, and experimental condition, *F*_(9, 225)_ = 5.40, *p* < 0.001, η^2^ = 0.18, were also significant. The RTs for AT, AVT-c and AVT-ic stimuli, but not the VT stimuli, were significantly faster in the monitored than in the alone experimental conditions (for all pairwise *post hoc* comparisons *p* < 0.05). In the monitored experimental condition, mean RTs for the congruent and incongruent multisensory conditions were not affected by switching between the target stimuli; Only the RTs to unisensory AT and VT stimuli slowed significantly when switching between unisensory stimuli (*p* < 0.01 for all *post-hoc* pairwise comparisons). In the alone experimental conditions, on the other hand, mean RTs for both the congruent and incongruent multisensory stimuli slowed down when switching from the unisensory AT stimulus (*p* < 0.01); when switching from AT, the increase in RTs was significantly greater for the congruent AVT-c than the incongruent AVT-ic stimulus (see Figure 3A).

Participants' RT variability also increased in the alone as compared to the monitored condition, *F*_(1, 25)_ = 6.95, *p* = 0.01, η^2^ = 0.22. The RT coefficient of variation was also significantly higher for the unisensory than for the incongruent multisensory stimuli, *F*_(3, 225)_ = 18.50, *p* < 0.001, η^2^ = 0.43, and *p* < 0.01, for all *post hoc* main effects comparisons. The main effect of switch condition was also significant, *F*_(9, 225)_ = 5.40, *p* < 0.001, η^2^ = 0.18, with switching from VT stimuli resulting in a significantly higher *Cv* than the AT and AVT-c switch conditions (*p* < 0.02). The interaction between stimulus type and switch condition was also significant, *F*_(9, 225)_ = 6.50, *p* < 0.001, η^2^ = 0.21. For AT stimuli, switching from a unisensory VT stimulus resulted in an increase in RT variability (*p* < 0.01). However, switching did not affect the RT variability for the other stimuli significantly (see Figure 3B).

An increase in RT variability across individuals can also be observed in the moving averages that are presented in Figure 4A. There is much more *SEM* overlap between the different stimuli in the alone than the monitored condition throughout the entire testing phase. Consistent with the results of Experiment 1, the variability across participants was analyzed revealing a significant increase in the alone condition, which was significantly higher in the switch than in the repeat condition (see Appendix C).

### Switch Costs

Consistent with the results of Experiment 1, switch costs were greatest when switching between unisensory stimuli (see Figure 4B). A two-way ANOVA revealed a significant main effect of switch, *F*_(11, 275)_ = 17.41, *p* < 0.001, η^2^ = 0.41, and experimental condition, *F*_(1, 25)_ = 9.71, *p* = 0.005, η^2^ = 0.28. Switch costs were significantly higher in the alone than in the monitored condition, and when switching between the unisensory AT and VT stimuli than for the multisensory stimuli. The interaction between the experimental and switch conditions was also significant, *F*_(11, 275)_ = 2.95, *p* = 0.001, η^2^ = 0.11. In the monitored condition, only switching between unisensory stimuli resulted in significantly higher switch costs (*p* < 0.05). In the alone condition, on the other hand, switching between AT and AVT-c stimuli also resulted in increased switch costs as compared to the other multisensory switch conditions (*p* < 0.01).

### Multisensory Enhancements

Multisensory enhancements for both switch and repeat conditions were affected by the experimental condition (see Figure 4C). In particular, multisensory enhancements were significantly higher for the switch than for the repeat conditions, *F*_(1, 25)_ = 84.04, *p* < 0.001, η^2^ = 0.77. The interaction between stimulus congruence and experimental condition was also significant, *F*_(1, 25)_ = 3.11, *p* = 0.01, η^2^ = 0.23. In the alone experimental conditions, there was no multisensory motor enhancement for congruent stimuli; multisensory gains were significantly higher for AVT-ic than for AVT-c stimuli (*p* = 0.002). In the monitored condition, there were no significant differences between the congruent and the incongruent stimuli (*p* > 0.5).

**Figure 5:**
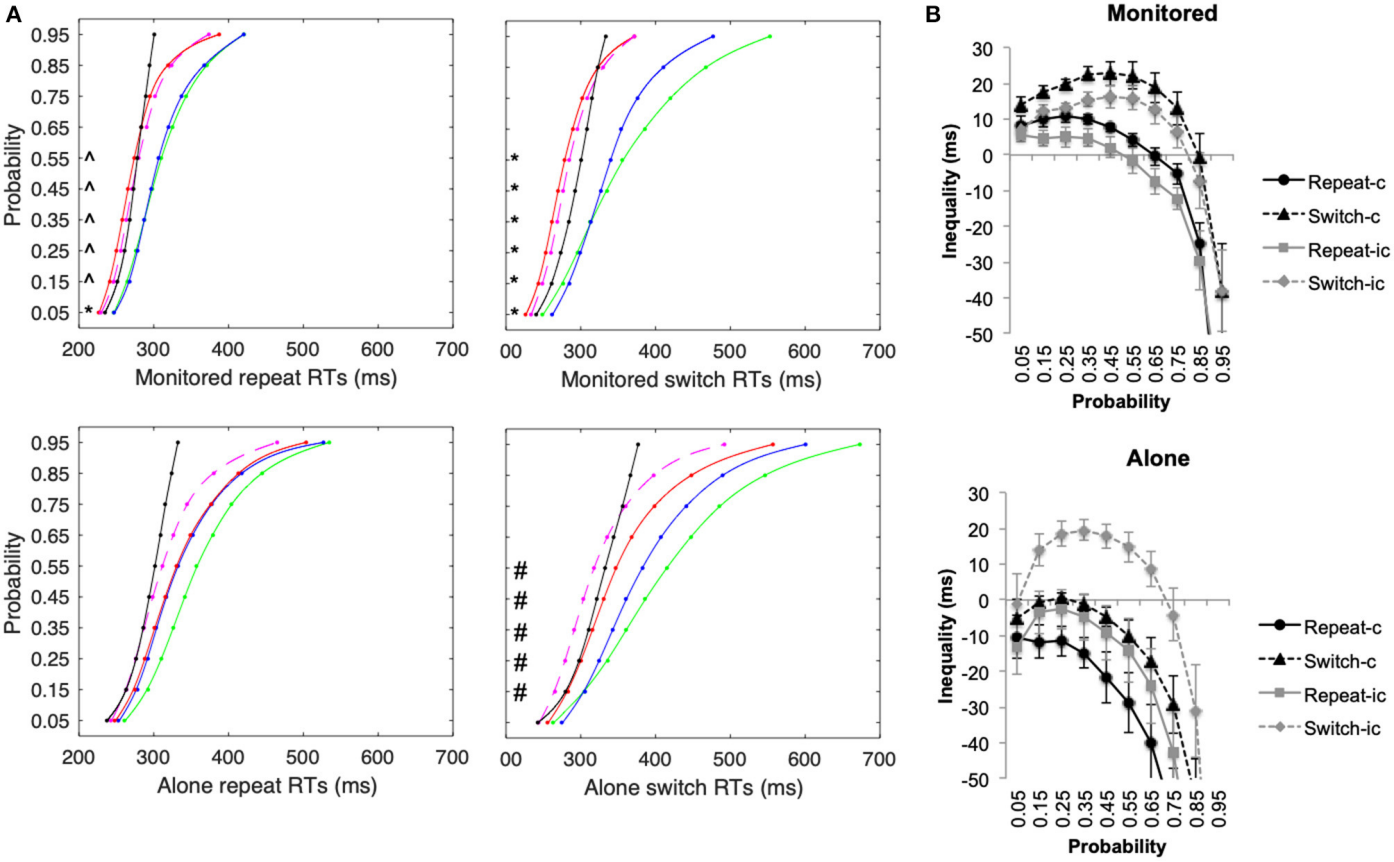
Experiment 2 **(A)** Cumulative density functions (CDFs) for the unisensory auditory (green line), visual (blue line) stimuli, and the multisensory congruent (AV-c: red line) and incongruent (AV-ic: dashed magenta line) and the summed probability for the unisensory stimuli (AT+VT CDF—i.e., test of inequality: black line) for repeat and switch conditions. **(B)** Mean difference (±SEM) between the AVT CDF and the bound AT+VT CDFs for repeat and switch conditions for congruent and incongruent trials. *both AVT-c and AVT-ic significantly faster than AT+VT CDF, ^∧^only AVT-c significantly faster than AT+VT CDF, ^#^only AVT-ic faster than AT+VT CDF.

A similar pattern of results was observed when assessing Miller's test of inequality (see Figure 5). In the alone condition, there were no significant violations of Miller's inequality for repeat trials (see Figure 5A): congruent, *F*_(9, 135)_ = 30.16, *p* < 0.001, η^2^ = 0.67, and incongruent, *F*_(9, 135)_ = 21.13, *p* < 0.001, η^2^ = 0.59, stimuli. For switch trials in the alone condition, violations were observed only for multisensory incongruent stimuli starting at 0.15 probability, *F*_(1, 135)_ = 18.95, *p* < 0.001, η^2^ = 0.56. There were no significant violations of Miller's inequality for congruent switch trials, *F*_(1, 135)_ = 33.49, *p* < 0.001, η^2^ = 0.69 (Note that for the alone experimental condition, the significant F-statistics represent faster AT+VT CDFs at the slower end of the probability function).

In the monitored condition, by contrast, violations of Miller's test of inequality were observed for all congruency and switch conditions: repeat congruent, *F*_(9, 90)_ = 34.46, *p* < 0.001, η^2^ = 0.78, repeat incongruent, *F*_(9, 90)_ = 23.62, *p* < 0.001, η^2^ = 0.70, switch congruent, *F*_(9, 90)_ = 16.42, *p* < 0.001, η^2^ = 0.62, and switch incongruent *F*_(9, 90)_ = 17.20, *p* < 0.001, η^2^ = 0.63. In the monitored condition, RTs were significantly faster than the bound added probabilities of the unisensory conditions for all conditions except the fastest RTs at 0.05 probabilities for AVT-ic stimuli. Note that the level of inequality (as calculated and presented in Figure 5B) was significantly higher in the monitored condition than in the alone conditions for congruent repeat and switch conditions (*p* < 0.006), but not for incongruent letter stimuli (*p* > 0.09). This finding is consistent with the results of Experiment 1, where audiovisual stimuli with no prior learnt associations were used.

## Discussion

Consistent with the results of Experiment 1, switch costs were higher when switching between unisensory rather than between multisensory stimuli. Multisensory enhancements were dependent not only on letter congruence, but also on experimental conditions. In the monitored experimental conditions, multisensory motor enhancements were observed for both repeat and switch conditions. However, in the alone condition, multisensory enhancements were observed for incongruent letters, but not for congruent repeating stimuli. There is a complex interplay between multisensory motor enhancements, sensory switching, and letter congruence, which is shown here to be modulated by the presence of an experimenter in the testing room.

Consistent with prior studies, both familiar (letters) and arbitrarily paired novel (red circle and tone) multisensory stimuli resulted in motor enhancements, even when the multisensory stimulus pairs were incongruent with prior learning (e.g., Miller, 1982, 1991; Giray and Ulrich, 1993; Barutchu et al., 2009). In both experiments, moving averages suggest that the enhancement in motor speed is present from the initiation of the task and that this enhancement is maintained throughout the course of the tasks. Using an object discrimination task, Molholm et al. (2004) showed that only congruent multisensory objects with dual targets result in multisensory enhancement, while incongruent stimuli resulted in slower RTs, particularly when the visual signal was irrelevant and the sound was a relevant target (also see Barutchu et al., 2013b). These findings suggest that multisensory processes are primed to dual multisensory targets by the task instructions prior to the initiation of the task. Neural mechanisms can indeed be modulated by cues and other task instructions prior to stimulus onset and response initiation (e.g., Corbetta et al., 1993, 2000; Kastner et al., 1999; Ruz and Nobre, 2008; Stokes et al., 2009; Nobre and van Ede, 2018). Prioritizing multisensory processing to stimuli with multiple target components may be one of the ways in which the multisensory system maintains flexibility to cope with a multisensory environment that is in a constant state of flux.

Under monitored experimental conditions, there were no differences in the level of multisensory motor enhancements for congruent and incongruent stimuli. Interestingly, however, when the participants were left alone, multisensory enhancements were observed for the switch conditions and only for the incongruent repeat trials. The presence of an experimenter in the testing environment may have increased obedience to task instructions, vigilance, motivation and joint attention and, in turn, up-regulated multisensory integration to all relevant target stimuli irrespective of stimulus congruence (e.g., Putz, 1975; Wahn et al., 2017). Therefore, when attention is engaged, prior learnt associations could be vetoed to prioritize the task-relevant stimuli. Another possible explanation is that under attentionally-demanding conditions (i.e., in the monitored condition), top-down inputs that identify target multisensory stimuli, may have primed the multisensory system into early integration prior to the detection of the letters' incongruence. In contrast, under low attention conditions (i.e., alone condition), multisensory enhancements were not observed for repeat congruent stimuli. Instead, only the novel incongruent stimuli resulted in multisensory enhancements under switch conditions. In this case, the novel pairing of the incongruent letters is likely to be salient and capture attention, modulating multisensory processing, and thus leading to higher levels of multisensory enhancement. There is much overlap between the multisensory and attention neural networks, both of which include the posterior parietal cortex (PPC), frontal cortical brain regions, and the superior colliculus (e.g., Stein and Meredith, 1993; Driver and Noesselt, 2008; Andersen et al., 2009; Zuanazzi and Noppeney, 2019). Future studies need to investigate how these networks are influenced by social factors, like experimenter presence.

## General Discussion

To the best of our knowledge, this is the first study to investigate: (1) the interplay between multisensory processing, sensory switching, and stimulus congruence; and (2) report a replicable (as it happens, incidental) finding demonstrating how experimental conditions, such as having the experimenter in the room, can influence these complex relationships. Across two experiments, multisensory enhancements have consistently been shown to be higher under switch than repeat conditions. Our results also demonstrate that significant multisensory enhancements are observable under repeat conditions depending on the stimulus type and the experimental condition. When participants are left alone in the room, they are less likely to show multisensory enhancements for repeating and familiar congruent stimuli. The presence of an experimenter in the room significantly enhances multisensory processing.

The presence of an experimenter in the testing room is likely to influence joint attention, motivation, conformity, and obedience to task instructions, which have been shown to influence various perceptual decision processes (e.g., Sherif, 1935; Asch, 1956; Milgram, 1965; Putz, 1975; Atmaca et al., 2011; Dittrich et al., 2017). Indeed, multisensory processes are partly modulated by mechanisms of attention (e.g., Talsma et al., 2010) and joint attention (Wahn et al., 2017). Therefore, even though the experimenter was not visible to the participants in the monitored conditions, participants may have engaged in “joint attention” with the experimenter by assuming that the experimenter was also fixated on the stimuli being presented on the screen. Our findings are also consistent with those studies showing that task performance can be improved by the supervision and monitoring of participants (Putz, 1975; Gomez and Sanson, 1994), leading to “social facilitation.” Given the ease of the task, we assume that attention was upregulated in the presence of the experimenter, acting as the essential glue needed to bind multisensory signals outside the predictive bounds of race models. Further research may therefore be required in order to assess how the “attention load,” the difficulty of the multisensory task, and other social factors influence the effects of experimenter presence (e.g., the experimenter's evaluative attitude, direction of gaze, visibility, social presence, etc.).

Interestingly, in Experiment 2, the absence of the experimenter had the greatest impact on responses to congruent multisensory stimuli. In the alone condition, multisensory enhancements were only observed for the unfamiliar, novel audiovisual combinations (i.e., used in Experiment 1) and the incongruent letters (Experiment 2), but not the repeating congruent letters (Experiment 2). These interactions between stimulus congruence and switch costs cannot be entirely explained by general increases in arousal and social facilitation (with the presence of the experimenter) that would improve overall task performance. Consistent with Barutchu et al. (2018), similar multisensory enhancements were demonstrated for semantically congruent and incongruent multisensory stimuli under monitored experimental conditions. When participants are left alone in the experimental testing room, one is less likely to observe multisensory enhancements. In addition, in the alone conditions, switch costs are higher for congruent multisensory stimuli when switching from an auditory stimulus. Familiar stimuli like letters are more likely to be ignored if not attended, unlike novel combinations of incongruent audiovisual stimuli, which tend to automatically up-regulate attention and related neural processes. However, it is interesting to note that switch costs were higher for congruent letters, but only when switching from the auditory stimulus in the alone condition (i.e., when switching from phonemes to graphemes). In the present study, there may be a secondary unintentional task switch between speech and reading under low attention conditions, with the visual reading task dominating perception (Lukas et al., 2010; Kreutzfeldt et al., 2015). This observed multisensory switch-cost for congruent letters may also be related to the “switch-cost paradox” whereby switching from a difficult (speech perception) to a relatively easy (reading) task results in a greater switch costs even in the absence of an actual task switch as long as the switching stimuli are associated with tasks of relatively different levels of difficult (Barutchu et al., 2013a). Audiovisual stimulation (i.e., being able to visualize lip movements) is known to enhance auditory speech perception (e.g., Sumby and Pollack, 1954; Raij et al., 2000; Ma et al., 2009). In the alone condition, assuming that participants were paying less attention, the congruent audiovisual letters may have been easier to process than the auditory only phonemes leading to a switch-cost paradox (i.e., higher switch costs for congruent letters following a AT switch). The low switch costs between visual and congruent audiovisual letters, and auditory and incongruent audiovisual letters, may reflect the fact that they are more similar to each other in processing difficulty. Further research is needed in order to investigate whether this switch cost for congruent audiovisual letters generalizes to other familiar stimuli (e.g., animals or other common objects), and how these relationships are modulated by attention and task difficulty.

Although multisensory enhancements were observed for both repeat and switch conditions throughout the entire sequence of trials (as observed in the moving averages), switching between unisensory stimuli resulted in slower and more variable RTs than repeat trials (e.g., Cohen and Rist, 1992; Turatto et al., 2002; Otto and Mamassian, 2012, 2016; Shaw et al., 2020). RTs to multisensory stimuli, on the other hand, did not slow down significantly following a stimulus switch; Switch costs were very low for multisensory stimuli, which may be explained by the fact that when switching from a unisensory to a multisensory stimulus either the auditory or the visual signal always repeat, thus eliminating the switch effect. In this study switching was defined by a sequential change in stimulus type, and since switch costs are higher for unisensory than multisensory stimuli, naturally this amplifies the observed multisensory motor enhancement for switching stimuli (i.e., as only the RTs for the unisensory stimuli, but not the multisensory stimuli, slow down following a switch). However, significant multisensory enhancements were still observed for repeat conditions, thus suggesting that sensory switches contribute, but cannot entirely explain, multisensory motor enhancements on their own.

Another proposed explanation for such multisensory motor speed enhancements is that they may relate to an increase in variability in response to multisensory signals when the decision is contingent on the detection of both sensory signals (Otto and Mamassian, 2012; Otto et al., 2013). This hypothesis has received support when comparing RTs to multisensory stimuli from a simple detection task (i.e., respond to all signals as in the classic redundant signal paradigm) to RTs from a discrimination task (i.e., respond only to multisensory signals and ignore unisensory stimuli in order to ensure that both sensory signals are detected before a response is elicited). However, in the studies by Otto and colleagues, the observed increase in mean RTs, and in the variability of RTs, could also be explained by the fact that RTs from a simple detection task were compared to RTs from a discrimination task requiring the suppression or inhibition of responses to unisensory signals. Based on this rationale, one would still have expected an increase in RT variability for multisensory stimuli even in the detection paradigm used here. However, in contrast, our results demonstrate that the variability of RTs for multisensory stimuli remains relatively constant for both switch and repeat conditions. These findings are consistent with those of Downing et al. (2014) who used a discrimination task with distractors to demonstrate that the RT variability for multisensory stimuli remained below that for unisensory stimuli even with added multisensory distractors. Therefore, in the present study, the increase in the RT variability at an individual level to multisensory stimuli cannot explain the observed multisensory gains under repeat and switch conditions.

Interestingly, we also observed a significant increase of RT variability across individuals when the participants were left alone in the testing room; RT variability is affected both within and across individuals. This finding was replicated in a second experiment (see also Appendix C). In Experiment 1, although mean RTs and the coefficient of variability of RTs did not differ significantly between the alone and monitored conditions, we observed a higher standard error of the mean (SEM) across individuals. In Experiment 2, we observed an increase in RT variability both, within and across individuals, which may be explained by the fact that in Experiment 2 familiar stimuli were used. This was the case even when the mean RTs showed significant motor enhancements, which suggests caution against relying on the mean RTs when evaluating multisensory gains. An increase in fidgeting and subtle changes in eye moments in the alone condition may have led to a reduction in motor stability; this should to be investigated by future studies using eye trackers or electrooculograms (EOGs), for example, to understand how such alternative monitoring approaches affect participants' performance in multisensory tasks. An increase in motor variability is likely to reduce statistical power and the likelihood of finding a significant difference in the RT distribution. Thus, differences between the present study and previous research may be related to differences in testing conditions (Otto and Mamassian, 2012; Otto et al., 2013), as race-model violations are more likely to be observed when the experimenter is present in the testing room.

In the present study, participant numbers for each experiment were determined using power analyses assuming moderate effect sizes. While acknowledging that it was not the original aim of this study to investigate the incidental finding of “social influences” on multisensory processes, even with our small numbers of participants, we are able to reliably show the effects of stimulus congruence, sensory switching, and experimenter presence (and their interactions) on multisensory processing. Our participant numbers are comparable to past studies in the field with participant numbers as low as 10 (e.g., Otto et al., 2013). Using a new approach, we have demonstrated that the non-significant effects between our groups are due to significant increases in within-group variability across participants in the alone condition, which, in turn, reduced the effect size and power of our statistical tests (see Appendix C). Future studies may want to consider replicating the study with higher participant numbers to investigate more subtle social influences on multisensory processes.

In conclusion, sensory switching has a greater cost when switching between unisensory than when switching between multisensory stimuli, inflating multisensory enhancements under sensory switch conditions. Nevertheless, we also observed faster RTs for repeating multisensory stimuli, which were partly dependent on stimulus novelty, letter congruence and social factors, such as having the experimenter present in the room. Motor enhancements are more likely to be observed for novel audiovisual combinations with the experimenter seated in the room. Further research is needed to investigate how social factors associated with the presence of an experimenter influence attention, motivation, conformity, and obedience to task instruction during multisensory processing.

## Data Availability Statement

The raw data supporting the conclusions of this article will be made available by the authors, without undue reservation.

## Ethics Statement

The studies involving human participants were reviewed and approved by Inter Divisional Medical Sciences Research Ethics Committee, University of Oxford. The participants provided their written informed consent to participate in this study.

## Author Contributions

AB contributed to experimental design, data collection, data analysis, and write-up of the manuscript. CS contributed to data analysis, interpretation, and the write-up of the manuscript. Both authors contributed to the article and approved the submitted version.

## Conflict of Interest

The authors declare that the research was conducted in the absence of any commercial or financial relationships that could be construed as a potential conflict of interest.

## References

[B1] Alsius A. Navarra J. Campbell R. Soto-Faraco S. (2005). Audiovisual integration of speech falters under high attention demands. Curr. Biol. 15, 839–843. 10.1016/j.cub.2005.03.046 15886102

[B2] Andersen T. S. Tiippana K. Laarni J. Kojo I. Sams M. (2009). The role of visual spatial attention in audiovisual speech perception. Speech Commun. 51, 184–193. 10.1016/j.specom.2008.07.004

[B3] Asch S. E. (1956). Studies of independence and conformity: I. A minority of one against a unanimous majority. Psychol. Monogr. 70, 1–70. 10.1037/h0093718

[B4] Atmaca S. Sebanz N. Knoblich G. (2011). The joint flanker effect: sharing tasks with real and imagined co-actors. Exp. Brain Res. 211, 371–385. 10.1007/s00221-011-2709-9 21573746 PMC3102196

[B5] Barutchu A. Becker S. I. Carter O. Hester R. Levy N. L. (2013a). The role of task-related learned representations in explaining asymmetries in task switching. PLoS ONE 8:e61729. 10.1371/journal.pone.0061729 23613919 PMC3628671

[B6] Barutchu A. Crewther D. P. Crewther S. G. (2009). The race that precedes coactivation: development of multisensory facilitation in children. Dev. Sci. 12, 464–473. 10.1111/j.1467-7687.2008.00782.x 19371371

[B7] Barutchu A. Freestone D. R. Innes-Brown H. Crewther D. P. Crewther S. G. (2013b). Evidence for enhanced multisensory facilitation with stimulus relevance: an electrophysiological investigation. PLoS ONE 8:e52978. 10.1371/journal.pone.0052978 23372652 PMC3553102

[B8] Barutchu A. Sahu A. Humphreys G. W. Spence C. (2019a). Multisensory processing in event-based prospective memory. Acta Psychol. 192, 23–30. 10.1016/j.actpsy.2018.10.015 30391627

[B9] Barutchu A. Spence C. Humphreys G. W. (2018). Multisensory enhancement elicited by unconscious visual stimuli. Exp. Brain Res. 236, 409–417. 10.1007/s00221-017-5140-z 29197998 PMC5809521

[B10] Barutchu A. Toohey S. Shivdasani M. N. Fifer J. M. Crewther S. G. Grayden D. B.. (2019b). Multisensory perception and attention in school-age children. J. Exp. Child Psycholol. 180, 141–155. 10.1016/j.jecp.2018.11.021 30655099

[B11] Belletier C. Camos V. (2018). Does the experimenter presence affect working memory? Ann. N. Y. Acad. Sci. 1424, 212–220. 10.1111/nyas.13627 29524358

[B12] Belletier C. Davranche K. Tellier I. S. Dumas F. Vidal F. Hasbroucq T.. (2015). Choking under monitoring pressure: being watched by the experimenter reduces executive attention. Psychon. Bull. Rev. 22, 1410–1416. 10.3758/s13423-015-0804-9 25673216

[B13] Botta F. Santangelo V. Raffone A. Sanabria D. Lupianez J. Belardinelli M. O. (2011). Multisensory integration affects visuo-spatial working memory. J. Exp. Psychol. Hum. Percept. Perform. 37, 1099–1109. 10.1037/a0023513 21553989

[B14] Bremner A. J. Lewkowicz D. J. Spence C. (Eds.). (2012). Multisensory Development. Oxford: Oxford University Press.

[B15] Chen Y.-C. Spence C. (2010). When hearing the bark helps to identify the dog: semantically-congruent sounds modulate the identification of masked pictures. Cognition 114, 389–404. 10.1016/j.cognition.2009.10.012 19909945

[B16] Chen Y.-C. Spence C. (2013). The time-course of the cross-modal semantic modulation of visual picture processing by naturalistic sounds and spoken words. Multisens. Res. 26, 371–386. 10.1163/22134808-00002420 24319929

[B17] Chen Y.-C. Spence C. (2017). Assessing the role of the 'unity assumption' on multisensory integration: a review. Front. Psychol. 8:445. 10.3389/fpsyg.2017.00445 28408890 PMC5374162

[B18] Chen Y.-C. Spence C. (2018). Dissociating the time courses of the cross-modal semantic priming effects elicited by naturalistic sounds and spoken words. Psychon. Bull. Rev. 25, 1138–1146. 10.3758/s13423-017-1324-6 28600716 PMC5990551

[B19] Cohen R. Rist F. (1992). The modality shift effect. Further explorations at the crossroads. Ann. N. Y. Acad. Sci. 658, 163–181. 10.1111/j.1749-6632.1992.tb22844.x 1497257

[B20] Corbetta M. Kincade J. M. Ollinger J. M. McAvoy M. P. Shulman G. L. (2000). Voluntary orienting is dissociated from target detection in human posterior parietal cortex. Nat. Neurosci. 3, 292–297. 10.1038/73009 10700263

[B21] Corbetta M. Miezin F. M. Shulman G. L. Petersen S. E. (1993). A PET study of visuospatial attention. J. Neurosci. 13, 1202–1226. 10.1523/JNEUROSCI.13-03-01202.1993 8441008 PMC6576604

[B22] Cox D. Hong S. W. (2015). Semantic-based crossmodal processing during visual suppression. Front. Psycholol. 6:722. 10.3389/fpsyg.2015.00722 26082736 PMC4451233

[B23] Cumming G. (2013). Understanding the New Statistics. New York, NY: Routledge.

[B24] Delogu F. Raffone A. Belardinelli M. O. (2009). Semantic encoding in working memory: is there a (multi)modality effect? Memory 17, 655–663. 10.1080/09658210902998054 19536688

[B25] Dittrich K. Bossert M. L. Rothe-Wulf A. Klauer K. C. (2017). The joint flanker effect and the joint Simon effect: on the comparability of processes underlying joint compatibility effects. Q. J. Exp. Psychol. 70, 1808–1823. 10.1080/17470218.2016.1207690 27357224

[B26] Downing H. C. Barutchu A. Crewther S. G. (2014). Developmental trends in the facilitation of multisensory objects with distractors. Front. Psychol. 5:1559. 10.3389/fpsyg.2014.01559 25653630 PMC4298743

[B27] Driver J. Noesselt T. (2008). Multisensory interplay reveals crossmodal influences on ‘sensory-specific’ brain regions, neural responses, and judgments. Neuron 57, 11–23. 10.1016/j.neuron.2007.12.013 18184561 PMC2427054

[B28] Fairhall S. L. Macaluso E. (2009). Spatial attention can modulate audiovisual integration at multiple cortical and subcortical sites. Eur. J. Neurosci. 29, 1247–1257. 10.1111/j.1460-9568.2009.06688.x 19302160

[B29] Faul F. Erdfelder E. Lang A. G. Buchner A. (2007). G^*^Power 3: a flexible statistical power analysis program for the social, behavioral, and biomedical sciences. Behav. Res. Methods 39, 175–191. 10.3758/BF03193146 17695343

[B30] Ferstl R. Hanewinkel R. Krag P. (1994). Is the modality-shift effect specific for schizophrenia patients? Schizophr. Bull. 20, 367–373. 10.1093/schbul/20.2.367 8085138

[B31] Fifer J. M. Barutchu A. Shivdasani M. N. Crewther S. G. (2013). Verbal and novel multisensory associative learning in adults. F1000Res 2:34. 10.12688/f1000research.2-34.v2 24627770 PMC3907154

[B32] Flom R. Bahrick L. E. (2010). The effects of intersensory redundancy on attention and memory: infants' long-term memory for orientation in audiovisual events. Dev. Psychol. 46, 428–436. 10.1037/a0018410 20210501 PMC2897054

[B33] Gau R. Noppeney U. (2016). How prior expectations shape multisensory perception. Neuroimage 124, 876–886. 10.1016/j.neuroimage.2015.09.045 26419391

[B34] Giard M. H. Peronnet F. (1999). Auditory-visual integration during multimodal object recognition in humans: a behavioral and electrophysiological study. J. Cogn. Neurosci. 11, 473–490. 10.1162/089892999563544 10511637

[B35] Giray M. Ulrich R. (1993). Motor coactivation revealed by response force in divided and focused attention. J. Exp. Psychol. 19, 1278–1291. 10.1037/0096-1523.19.6.1278 8294892

[B36] Gomez R. Sanson A. V. (1994). Effects of experimenter and mother presence on the attentional performance and activity of hyperactive boys. J. Abnorm. Child Psychol. 22, 517–529. 10.1007/BF02168935 7822626

[B37] Gregory S. E. Jackson M. C. (2017). Joint attention enhances visual working memory. J. Exp. Psychol. 43, 237–249. 10.1037/xlm0000294 27359225

[B38] Hanewinkel R. Ferstl R. (1996). Effects of modality shift and motor response shift on simple reaction time in schizophrenia patients. J. Abnorm. Psychol. 105, 459–463. 10.1037/0021-843X.105.3.459 8772017

[B39] Harrar V. Tammam J. Perez-Bellido A. Pitt A. Stein J. Spence C. (2014). Multisensory integration and attention in developmental dyslexia. Curr. Biol. 24, 531–535. 10.1016/j.cub.2014.01.029 24530067

[B40] Hobeika L. Taffou M. Viaud-Delmon I. (2020). Social impact on audiotactile integration near the body. Acoust. Sci. Technol. 41, 345–348. 10.1250/ast.41.345

[B41] Innes B. R. Otto T. U. (2019). A comparative analysis of response times shows that multisensory benefits and interactions are not equivalent. Sci. Rep. 9:2921. 10.1038/s41598-019-39924-6 30814642 PMC6393672

[B42] Kastner S. Pinsk M. A. De Weerd P. Desimone R. Ungerleider L. G. (1999). Increased activity in human visual cortex during directed attention in the absence of visual stimulation. Neuron 22, 751–761. 10.1016/S0896-6273(00)80734-5 10230795

[B43] Kreutzfeldt M. Stephan D. N. Sturm W. Willmes K. Koch I. (2015). The role of crossmodal competition and dimensional overlap in crossmodal attention switching. Acta Psycholol. 155, 67–76. 10.1016/j.actpsy.2014.12.006 25577489

[B44] Lin G. Carlile S. (2015). Costs of switching auditory spatial attention in following conversational turn-taking. Front. Neurosci. 9:124. 10.3389/fnins.2015.00124 25941466 PMC4403343

[B45] Liu Y. Otto T. U. (2020). The role of context in experiments and models of multisensory decision making. J. Math. Psychol. 96:102352 10.1016/j.jmp.2020.102352

[B46] Longman C. S. Lavric A. Munteanu C. Monsell S. (2014). Attentional inertia and delayed orienting of spatial attention in task-switching. J. Exp. Psychol. 40, 1580–1602. 10.1037/a0036552 24842065

[B47] Lukas S. Philipp A. M. Koch I. (2010). Switching attention between modalities: further evidence for visual dominance. Psychol. Res. 74, 255–267. 10.1007/s00426-009-0246-y 19517132

[B48] Ma W. J. Zhou X. Ross L. A. Foxe J. J. Parra L. C. (2009). Lip-reading aids word recognition most in moderate noise: a Bayesian explanation using high-dimensional feature space. PLoS ONE 4:e4638. 10.1371/journal.pone.0004638 19259259 PMC2645675

[B49] Milgram S. (1965). Behavioral study of obedience. J. Abnorm. Soc. Psychol. 67, 371–378. 10.1037/h0040525 14049516

[B50] Miller J. (1982). Divided attention: evidence for coactivation with redundant signals. Cogn. Psychol. 14, 247–279. 10.1016/0010-0285(82)90010-X 7083803

[B51] Miller J. (1986). Timecourse of coactivation in bimodal divided attention. Percept. Psychophys. 40, 331–343. 10.3758/BF03203025 3786102

[B52] Miller J. (1991). Channel interaction and the redundant-targets effect in bimodal divided attention. J. Exp. Psychol. 17, 160–169. 10.1037/0096-1523.17.1.160 1826309

[B53] Molholm S. Ritter W. Javitt D. C. Foxe J. J. (2004). Multisensory visual-auditory object recognition in humans: a high-density electrical mapping study. Cerebral Cortex 14, 452–465. 10.1093/cercor/bhh007 15028649

[B54] Nobre A. C. van Ede F. (2018). Anticipated moments: temporal structure in attention. Nat. Rev. Neurosci. 19, 34–48. 10.1038/nrn.2017.141 29213134

[B55] Otto T. U. Dassy B. Mamassian P. (2013). Principles of multisensory behavior. J. Neurosci. 33, 7463–7474. 10.1523/JNEUROSCI.4678-12.2013 23616552 PMC6619564

[B56] Otto T. U. Mamassian P. (2012). Noise and correlations in parallel perceptual decision making. Curr. Biol. 22, 1391–1396. 10.1016/j.cub.2012.05.031 22771043

[B57] Otto T. U. Mamassian P. (2016). Multisensory decisions: the test of a race model, its logic, and power. Multisens. Res. 30, 1–24. 10.1163/22134808-00002541

[B58] Putz V. R. (1975). The effects of different modes of supervision on vigilance behaviour. Br. J. Psychol. 66, 157–160. 10.1111/j.2044-8295.1975.tb01449.x 1156737

[B59] Raij T. Uutela K. Hari R. (2000). Audiovisual integration of letters in the human brain. Neuron 28, 617–625. 10.1016/S0896-6273(00)00138-0 11144369

[B60] Risko E. F. Kingstone A. (2011). Eyes wide shut: Implied social presence, eye tracking and attention. Atten. Percept. Psychophys. 73, 291–296. 10.3758/s13414-010-0042-1 21264723

[B61] Ruz M. Nobre A. C. (2008). Dissociable top-down anticipatory neural states for different linguistic dimensions. Neuropsychologia 46, 1151–1160. 10.1016/j.neuropsychologia.2007.10.021 18083202

[B62] Seitz A. R. Kim R. Shams L. (2006). Sound facilitates visual learning. Curr. Biol. 16, 1422–1427. 10.1016/j.cub.2006.05.048 16860741

[B63] Shams L. Seitz A. R. (2008). Benefits of multisensory learning. Trends Cogn. Sci. 12, 411–417. 10.1016/j.tics.2008.07.006 18805039

[B64] Shaw L. H. Freeman E. G. Crosse M. J. Nicholas E. Chen A. M. Brainman M. S.. (2020). Operating in a multisensory context: assessing the interplay between multisensory reaction time facilitation and inter-sensory task-switching effects. Neuroscience 436, 122–135. 10.1016/j.neuroscience.2020.04.013 32325100

[B65] Shepherdson P. Miller J. (2016). Non-semantic contributions to “semantic” redundancy gain. Q. J. Exp. Psychol. 69, 1564–1582. 10.1080/17470218.2015.1088555 26339718

[B66] Sherif M. (1935). A study of some social factors in perception. Arch. Psychol, 187, 1–60.

[B67] Sinnett S. Soto-Faraco S. Spence C. (2008). The co-occurrence of multisensory competition and facilitation. Acta Psychol. 128, 153–161. 10.1016/j.actpsy.2007.12.002 18207117

[B68] Soto-Faraco S. Kvasova D. Biau E. Ikumi N. Ruzzoli M. Morís-Fernández L.. (2019). Multisensory Interactions in the Real World. Cambridge: Cambridge University Press.

[B69] Spence C. (2013). Just how important is spatial coincidence to multisensory integration? Evaluating the spatial rule. Ann. N. Y. Acad. Sci. 1296, 31–49. 10.1111/nyas.12121 23710729

[B70] Spence C. Nicholls M. E. Driver J. (2001). The cost of expecting events in the wrong sensory modality. Percept. Psychophys. 63, 330–336. 10.3758/BF03194473 11281107

[B71] Spence C. Soto-Faraco S. (2020). Crossmodal Attention Applied: Lessons for/From Driving. Cambridge Elements of Attention. Cambridge: Cambridge University Press.

[B72] Stein B. E. Meredith M. A. (1993). The Merging of the Senses. Cambridge, MA: MIT Press.

[B73] Stokes M. Thompson R. Nobre A. C. Duncan J. (2009). Shape-specific preparatory activity mediates attention to targets in human visual cortex. Proc. Natl. Acad. Sci. U.S.A. 106, 19569–19574. 10.1073/pnas.0905306106 19887644 PMC2772815

[B74] Sumby W. H. Pollack I. (1954). Visual contribution to speech intelligibility in noise. J. Acoust. Soc. Am. 26, 212–215. 10.1121/1.1907309

[B75] Sutton S. Hakerem G. Zubin J. Portnoy M. (1961). The effect of shift of sensory modality on serial reaction-time: a comparison of schizophrenics and normals. Am. J. Psychol. 74, 224–232. 10.2307/1419407

[B76] Swainson R. Martin D. Prosser L. (2017). Task-switch costs subsequent to cue-only trials. Q. J. Exp. Psychol. 70, 1453–1470. 10.1080/17470218.2016.1188321 27174655

[B77] Talsma D. (2015). Predictive coding and multisensory integration: an attentional account of the multisensory mind. Front. Integr. Neurosci. 9:19. 10.3389/fnint.2015.00019 25859192 PMC4374459

[B78] Talsma D. Senkowski D. Soto-Faraco S. Woldorff M. G. (2010). The multifaceted interplay between attention and multisensory integration. Trends Cogn. Sci. 14, 400–410. 10.1016/j.tics.2010.06.008 20675182 PMC3306770

[B79] Turatto M. Benso F. Galfano G. Umilta C. (2002). Nonspatial attentional shifts between audition and vision. J. Exp. Psychol. 28, 628–639. 10.1037/0096-1523.28.3.628 12075893

[B80] Wahn B. Keshava A. Sinnett S. Kingstone A. König P. (2017). Audiovisual integration is affected by performing a task jointly, in Proceedings of the 39th Annual Conference of the Cognitive Science Society (Austin, TX), 1296–1301.

[B81] Zuanazzi A. Noppeney U. (2019). Distinct neural mechanisms of spatial attention and expectation guide perceptual Inference in a multisensory world. J. Neurosci. 39, 2301–2312. 10.1523/JNEUROSCI.2873-18.2019 30659086 PMC6433765

