# Supplementary material for: An Experimenter's Influence on Motor Enhancements: The Effects of Letter Congruency and Sensory Switch-Costs on Multisensory Integration
Source: Front Psychol. 2020 Dec 1;11:588343. doi: 10.3389/fpsyg.2020.588343 (PMC7736551; doi:10.3389/fpsyg.2020.588343)
Supplement: Supplementary file 1 [file Data_Sheet_1.pdf]

APPENDIX A

Experiment 1: Analysis of error rates

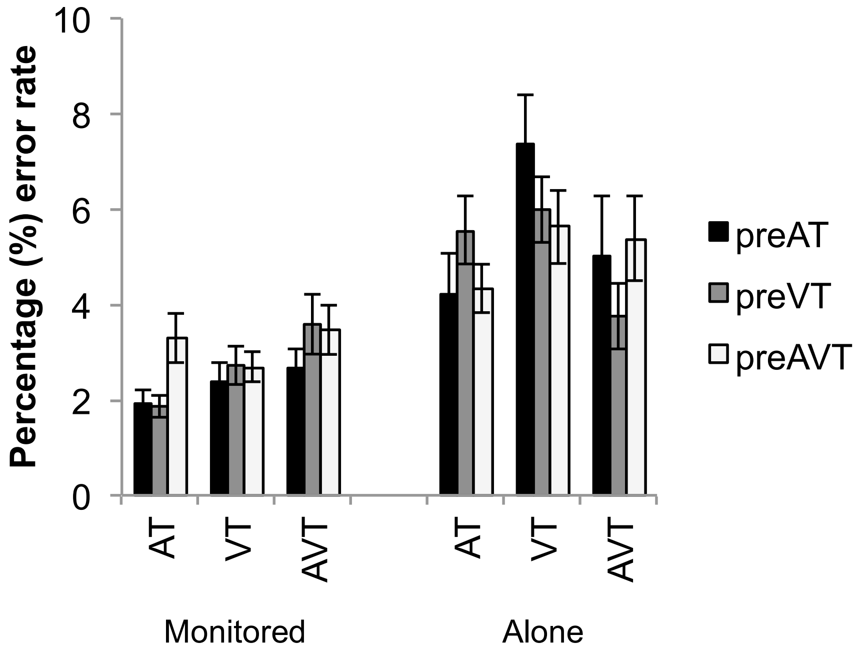

Figure S1. Experiment 1: Mean percentage error rate (+SEM) for the auditory (AT), visual (VT), and audiovisual (AVT) stimuli, switch (pre-AT, pre-VT, and pre-AVT) and experimental (monitored and alone) conditions.

As expected, the overall percentage of errors on the simple detection task was low, averaging below 10% mean errors for all stimulus conditions (Figure S1). Crucially, error rates were significantly lower when the experimenter was present in the room (M = 2.74%, SE = 0.74) than when the participants were left alone (M = 5.25%, SE = 0.47), $F(1,25) = 12.64$, $p = .002$, $\eta^2 = .34$, thus suggesting that the participants were more attentive in the former condition. The two-way interaction between testing condition and stimulus type, $F(2,100) = 4.11$, $p = .02$, $\eta^2 = .14$, and the three-way interaction between testing condition, stimulus type, and switch condition, $F(4,100) = 4.53$, $p = .002$, $\eta^2 = .15$, were also significant. Follow-up simple effects

analyses using pairwise comparisons revealed that there were no significant differences between the different switch conditions ($p > .05$). Error rates were significantly higher in the alone condition than in the monitored condition for the repeat AT stimulus ($p = .03$) and the AT stimulus following a switch from VT (i.e., $p = .001$), and for all VT stimuli and switch conditions ($p < .01$). However, the testing condition did not exert a significant influence over the error rates for the AVT stimuli and switch conditions ($p > .08$ for all). In the monitored condition, switching from VT resulted in significantly higher error rates for AT than AVT stimuli ($p = .03$). In the alone condition, however, error rates were significantly higher for VT stimuli preceded by an AT stimulus than an AT repeat stimulus (p = .005). Error rates were significantly lower for AVT stimuli than for the AT and VT stimuli preceded by a VT stimulus (p < .02). There were no other significant simple effects in the error data ($p >$. 05 for all).

APPENDIX B

Experiment 2: Analysis of error rates

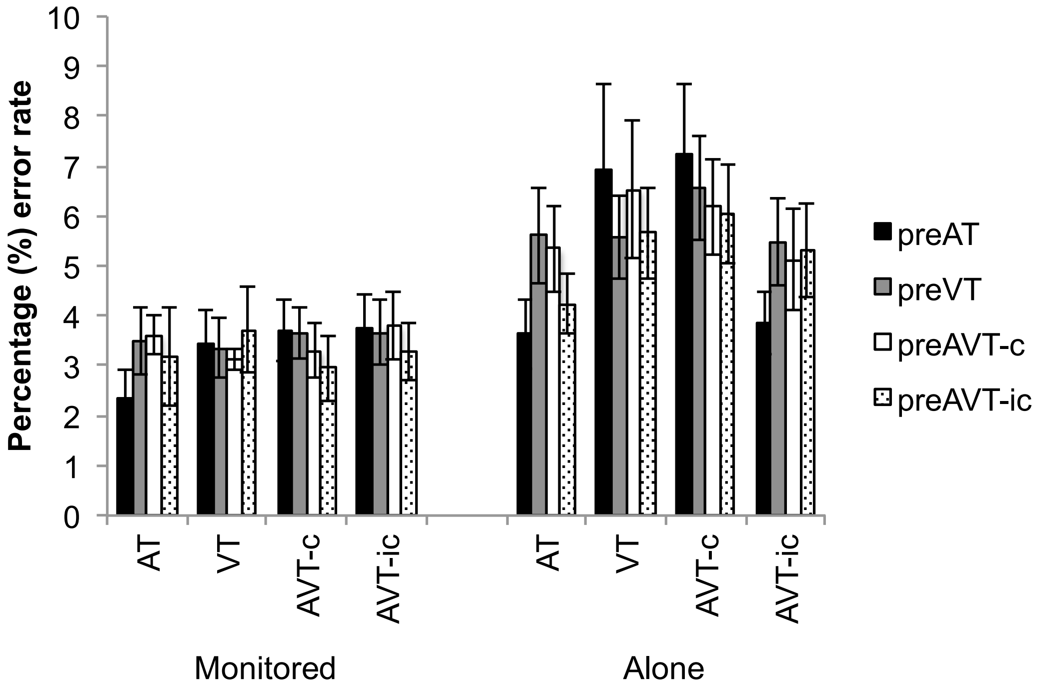

Figure S2. Experiment 2: Mean percentage error rates (±SEM) for auditory (AT), visual (VT), audiovisual congruent (AVT-c), and audiovisual incongruent (AVT-ic) stimuli and switch conditions.

The percentage of error rates averaged below 10% for all stimuli and experimental conditions (see Figure S2). A three-way ANOVA revealed a significant main effect of stimulus type, $F(3,225) = 2.79, p < .05, \eta^2 = .10$. Error rates were slightly, but significantly, higher for AVT-c then for AT stimuli ($p = .02$). There was a borderline significant main effect of experimental condition, $F(1,25) = 4.11, p = .05, \eta^2 = .09$; error rates were higher in the alone than in the monitored condition. The interactions between stimulus and experimental condition, $F(3,225) = 2.48$, $p = .07$, $\eta^2 = .09$, and stimulus by switch condition, $F(9,225) = 1.82$, $p = .07$, $\eta^2 = .07$, also approached significance. None of the other main or interaction effects approached significance ($p > .3$ for all).

Note that we re-ran the reaction time (RT) analyses with the alone and monitored conditions matched for accuracy, and we observe the same pattern of results.

APPENDIX C

In Experiments 1 and 2, the standard errors of the mean (*SEM*s) measures for RTs suggest that the variability across individuals was much higher in the alone than the monitored condition (see Figures 1, 3B, and 4). At present, there is no standard statistical test to assess whether such a change in variability across individuals within-groups is significant. Here we present a simple alternative. We calculated the 'absolute' difference between the overall 'group RT means' and the 'individual RT means' for repeat and switch trials (note that for this additional analysis we have collapsed all the repeat and switch trials to maintain simplicity and power). This provides a measure of individual RT variation from the group mean for repeat and switch trials in the alone and monitored conditions (see Table S1).

Table S1

Mean (+SEM) cross-group RT variations from group mean RTs for repeat and switch trials in the monitored and alone conditions for Experiments 1 and 2.

		Monitored		Alone	
		M	*SEM*	*M*	*SEM*
Exp1	Repeat	23.27	9.78	48.76	8.74
	Switch	25.44	11.71	61.93	10.47
Exp 2	Repeat	15.20	10.00	48.85	8.29
	Switch	16.95	10.59	57.24	8.78

For Experiments 1 and 2, a 2(switch and repeat) x 2(monitored and alone) mixed ANOVA was used to assess across group variations in RTs.

In Experiment 1, both main effects were significant; variability across participants was significantly higher in the alone condition, $F(1, 25) = 4.67$, $p = .04$, $\eta^2 = .16$, and in the switch condition, $F(1, 25) = 15.73$, $p < .001$, $\eta^2 = .39$. The interaction between switch type and monitoring condition was also significant, $F(1, 25) = 8.10$, $p = .009$, $\eta^2 = .25$. In the alone condition, stimulus switching resulted in significantly higher variability across individuals than the repeat conditions ($p < .001$). There were no significant differences between the switch and repeat trials in monitored condition.

In Experiment 2, both main effects were significant; variability across participants was significantly higher in the alone condition, $F(1, 25) = 5.23$, $p = .03$, $\eta^2 = .17$, and in the switch condition, $F(1, 25) = 12.22$, $p = .002$, $\eta^2 = .33$. The interaction between switch type and monitoring condition was also significant, $F(1, 25) = 5.23$, $p = .03$, $\eta^2 = .17$. In the alone condition, stimulus switching resulted in significantly higher variability across individuals than the repeat conditions ($p < .001$). There were no significant differences between the switch and repeat trials in the monitored condition.

The same pattern of increase in variability across the individuals within groups is observed in Experiments 1 and 2. Consistent with our observations of increased SEMs in the RTs (see Figures 1 and 4), within group RT variability significantly increases in the alone condition, with the increase being significantly greater in the switch condition conditions than the repeat conditions.

Importantly, this significant increase in within-group variability across individuals in the alone conditions helps to explain why the effect sizes are so low and non-significant for our ‘alone’ vs. ‘monitored’ group comparisons of RTs, including the race-violations.
